# Enhanced recovery after surgery: nursing strategy for total hip arthroplasty in older adult patients

**DOI:** 10.1186/s12877-025-05888-8

**Published:** 2025-04-25

**Authors:** Guifang Liu, Ling Li, Jiashuo Deng, Lin Cai, Ruolin He

**Affiliations:** 1 Department of Orthopedics, External hand trauma ward, Linquan County People’s Hospital, Fuyang City, 236400 Anhui province China; 2 Bengbu Medical University, Bengbu, 233030 Anhui Province China

**Keywords:** Hip function, Ability to daily life, VAS, Postoperative nursing, Hospitalization

## Abstract

**Background:**

The incidence of total hip arthroplasty (THA) is dramatically increasing, particularly in older adults. Enhanced recovery after surgery (ERAS) has been used in the postoperative care of patients undergoing surgical treatment.

**Aims:**

This study compared the effects of ERAS and regular nursing on older adult patients undergoing THA to evaluate ERAS’s potential in patients’ postoperative care.

**Methods:**

Ninety older adult patients (age ≥ 60 years) who underwent THA were enrolled and randomly divided into two groups: regular and ERAS nursing strategies. The ERAS nursing strategy was optimized based on regular nursing in terms of pain management, nutrition management, intestinal preparation, drainage tube nursing, catheter nursing, and normothermia maintenance. The efficiency of the two nursing strategies was evaluated from the perspectives of postoperative pain, hospitalization conditions, hip function, daily life ability, complications, and satisfaction.

**Results:**

The ERAS group showed earlier first aerofluxus, getting out of bed, and defecation; the moving distance after getting out of bed was greater than that in the regular group. The removal of urinary and drainage tubes was also earlier in the ERAS group than in the regular group. ERAS significantly alleviated postoperative pain, increased Harris scores and the Barthel index, reduced hospitalization duration and expenses, and lowered the occurrence of complications. The ERAS group also showed higher satisfaction levels than the regular group.

**Conclusions:**

This single-blind randomized controlled trial showed that the ERAS nursing strategy reduced pain, length and cost of hospital stay, and incidence of complications after THA compared with regular care. Therefore, ERAS nursing strategies are recommended to improve the postoperative recovery rates in older adult patients undergoing THA.

## Introduction

With the significant aging trend, the incidence of hip joint diseases, such as caput femoris necrosis and hip arthritis, is gradually increasing and has become one of the major etiologies of hospitalization in older adult patients, limiting patient mobility [1, 2]. With the development of artificial hip replacements, total hip arthroplasty (THA) has been widely employed in the clinical treatment of hip joint diseases, and the number of patients undergoing THA is increasing every year [3]. However, some problems persist in the clinical practice. First, the postoperative recovery period after THA is relatively longer and is accompanied by a risk of wound infection, muscle atrophy, joint dislocation, and other complications [4–6]. Additionally, the requirements for THA postoperative nursing are high, which demands that nursing staff possess sound and relevant knowledge [7]. The growing demand for THA promotes the need to develop targeted care programs that can not only accelerate the recovery of patients but also control costs, save medical resources, and improve medical quality and therapeutic efficiency.

Conventional care was provided according to fixed nursing procedures and standards, including disease observation, basic nursing, and health education. This nursing method often has the limitations of weak service awareness among nurses, poor service quality, insufficient basic care, and insufficient health education promotion, leading to poor pain management, slow recovery, and high complication rates [8–11]. Therefore, Enhanced Recovery After Surgery (ERAS) came into being [12]. The ERAS nursing strategy considers the patient at the center and optimizes the entire path before, during, and after surgery through the cooperation of surgery, anesthesia, nursing, nutrition, and other disciplines to achieve multi-win results. ‌ ERAS can significantly shorten the length of hospital stay, reduce complications, and improve patient satisfaction [13,14].‌ For example, in terms of pain management in THA patients, the ERAS group received multidisciplinary lectures and crutching-gait training before surgery, 90 mg of Etoricoxibe was administered as preemptive analgesia once an hour before surgery, and surgery was performed under spinal anesthesia with intravenous dexamethasone (8 mg). The control group received long-acting spinal anesthesia [13].

ERAS, also known as fast-track surgery (FTS), is based on evidence-based medicine, improving and optimizing perioperative treatments and postoperative nursing measures to reduce the traumatic stress of patients, and therefore promote rapid recovery and save medical resources [13]. ERAS can be subdivided into preoperative function optimization, preoperative education, blood management, pain, sleep management, fluid management, and other strategies [14]. The advantages of ERAS in the postoperative nursing of gastrointestinal surgery, hepatobiliary surgery, urology surgery, and other surgical emergency operations as well as in intensive care patients have been clinically confirmed [15–19]. Moreover, ERAS has shown advantages in gynecological oncology, pediatrics, and weight loss, which can shorten the length of hospital stay, reduce complications, and reduce costs [20]. Recently, ERAS has been applied in postoperative nursing after THA, and great progress has been made in shortening the postoperative hospital stay, shortening the recovery period, and achieving rapid functional recovery without increasing morbidity and mortality [21–23]. For example, Zhu et al. conducted a meta-analysis and reported that ERAS significantly reduced the postoperative length of stay (LOS) and complication rates in THA patients [24]. A meta-analysis conducted by Zhang et al. suggested that ERAS could significantly shorten the length of hospital stay, reduce the blood transfusion rate, and reduce mortality within 30 days after surgery without increasing postoperative complications and readmission rates in THA and TKA patients [25]. Another study suggested that a multidisciplinary team (MDT) nursing model based on the ERAS concept can effectively reduce postoperative pain, shorten the length of hospital stay, reduce hospitalization costs, and reduce the incidence of complications in perioperative THA/TKA patients [26]. However, efficiency and specification standards remain unclear, particularly in the remote areas of China.

Hence, this study evaluated the safety and efficiency of ERAS in the postoperative nursing of THA and investigated the satisfaction of older adult patients undergoing THA, aiming to explore adjustable factors, improve nursing strategies for ERAS, and provide a practical basis for its clinical promotion.

## Materials and methods

### Study participants

This study had been approved by the Ethics Committee of our hospital and enrolled 90 older adult patients who received THA at our Hospital during 2020–2023 according to the following criteria: (1) aging ≥ 60 years [27]; (2) primarily received unilateral THA; (3) signed informed consent. Patients with one of the following criteria were excluded: (1) history of hip surgery, (2) hip infection, (3) coagulation dysfunction or deep vein thrombosis, (4) operation time > 4 h, or (5) intraoperative blood transfusion > 500 mL [28–30].

### Sample size calculation and grouping

The sample size was calculated by comparing the mean values of two samples. The formula used is as follows:

$$\:\text{n}={({\text{Z}}_{{\upalpha\:}}+{\text{Z}}_{{\upbeta\:}})}^{2}\:\text{*}\:2{\sigma\:}^{2}/{\delta\:}^{2}$$

where, n represents the sample size of each group; set the test level α = 0.05, then Z_α_=1.96; Test efficiency β = 0.9, then Z_β_=1.28. According to the preliminary experiment of this study, the overall standard deviation σ was measured to be 6.95, and δ was the difference in mean between the ERAS group and the regular group, which was 5.11. The ratio of the ERAS group to the regular group was 1:1, and the statistic *n* ≈ 39. Considering a loss to follow-up rate of 15%, 90 patients were recruited.

All patients who met the inclusion criteria were divided into groups using the random number table method. First, the study participants were numbered from to 1–90. Second, starting from any row or column of the random number table, random numbers were read sequentially and recorded. Finally, all selected random numbers were sorted from smallest to largest, and the study participants were assigned to different groups. Allocation concealment: The randomly generated allocation sequence was placed in an envelope that was encoded, sealed, and opaque. Each envelope contained allocation information for a participant. Those who met the inclusion criteria were included in the study and numbered according to the order in which they joined the study. Simultaneously, the corresponding numbered envelopes were opened, and the patients were divided into ERAS and regular groups according to the grouping scheme in the envelope, with 45 patients in each group. Before opening the envelope, the researchers wrote the names and detailed information of the qualified participants on the surface of the envelope, which contained carbon paper to ensure the accuracy of the information. Assigning concealment ensured that each participant had an equal chance of being randomly assigned to each study group, avoiding selective bias and improving the reliability and scientificity of the trial. ‌‌‌Only the surgeon knew which group the patient was assigned to (single-blind). Additionally, the ERAS and regular groups were treated in different wards and on different floors to avoid contact and exchange.

### Staff training

Before the study, an ERAS nursing strategy group was established and group members were trained in ERAS-related theories and implementation methods. The training forms were online and offline. The main contents of the training were as follows: (1) conducting research program training for members of the research team and conducting training for researchers on the use of scales and precautions to ensure the smooth development of the research work. (2) Dietitians and rehabilitation therapists trained the members of the program and provided professional explanations of the content, key points, specific exercise times, and early postoperative exercise methods for physical rehabilitation training. (3) Surgeons were responsible for THA professional knowledge, training, and emergency handling. The head of the surgical nursing department was responsible for controlling the progress of the project. Surgical nurses and researchers were responsible for protocol implementation, health education, patient training, answering patient questions, and data processing. Only after group members were trained in the unified theory could those who cleared the assessment formally participate in the ERAS group.

### Nursing strategies

The nursing strategies of each group are summarized in Table 1. The medical and nursing teams in the two groups were the same.

**Table 1:** The nursing strategies of each group

		Regular group	ERAS group
Pain management	preoperative	On-demand pain relief	Preventive analgesia
	Intraoperative	Conventional anesthesia analgesia	Peripheral nerve block combined with the injection of a mixture of ropivacaine combined with adrenaline and glucocorticoid around the incision
	postoperative	Physical pain relief combined with oral/intravenous painkillers and patient controlled analgesia	Physical pain relief combined with oral/intravenous painkillers and patient controlled analgesia
Nutrition management	preoperative	regular diet	High-quality protein and an immune-boosting diet
	The night before surgery	Fast for 8–12 h and no drinking for 4–6 h	6–8 h before surgery: Avoiding solid foods and liquid protein 3–4 h before surgery: Avoiding carbohydrates; antiemetics; clear, non-alcoholic liquid rich in carbohydrates 2 h before surgery: Absolutely no drinking or fasting
	postoperative	Supine without the pillow and resume diet after the first exhaust	High pillow (head height of 40–50°) and high feet (foot height of 30°); Preventive application of antiemetics; An early diet with high-quality protein, high fiber, and an immune-boosting diet
Intestinal preparation	preoperative	Conventional enema	No intestinal preparation; for constipation patients to give an auxiliary defecation
Drainage tube nursing	Removing criteria	the 24 h drainage volume was less than 50 mL on the 2nd -3rd day after surgery	No obvious bleeding or serum separation in the drainage The drainage tube should be removed as soon as possible
Catheter nursing	Removing criteria	Removed on the 2nd -3rd day after surgery	Removed at the end of the operation; Indwelling time no more than 24 h for the patients need to be indwelled
Normothermia maintaining		Checking bode temperature four times a day until 3 days after the surgery	Preoperative: room temperature was adjusted to 25 °C half an hour before the patients entered operating room; Intraoperative: strengthen coverage, avoiding unnecessary exposure combined with heating measures; Postoperative: the liquid was heated with the infusion heating device

Patients could be discharged if they met the following criteria: (1) stable vital signs, (2) ability to use a waking frame or crutches to walk alone, (3) no postoperative complications, and (4) ability to get out of bed independently.

### Harris hip score

The hip function of the patients was evaluated using 4 items: pain (44 points), function (47 points), malformation (4 points), and range of motion (5 points) according to the Harris hip function scale [31]. The higher the score, the better the patients’ hip function. The Harris hip scores of the two groups of patients were recorded at two weeks, one month, and three months after surgery.

### Visual analog scale (VAS)

A total of 5 degrees were set in the VAS, including painless (0), mild pain (1–3), moderate pain (4–6), severe pain (7–9), and excruciating pain (10). The visual analog scale (VAS) scores of the two groups of patients were recorded one day after surgery, on the day they primarily got out of bed, and on the day of discharge.

### Satisfaction survey

Patient satisfaction starts from the first visit to the inpatient services and continues throughout the treatment and postoperative periods. A subjective questionnaire was administered to evaluate patient satisfaction. On the day of discharge, an anonymous questionnaire survey was administered to the patients as an indicator of postoperative satisfaction. This evaluation is based on a survey of 16 items, including “my physiotherapist was kind”; “my physiotherapist took care of my privacy”; “my physiotherapist gave me enough time”; “service secretary was helpful”; “I completed my secretarial procedures easily and early”; “the room was properly prepared, and it was warm sufficiently”; “the room, bed linen, and pillowcases were clean”; “hospital staff obeyed the hygiene rules”; “I was able to contact my doctor easily”; “my doctors’ explanations were sufficient and understandable”; “my doctor listened to me and answered my questions at enough time”; “my nurses were friendly during my treatment”; “my nurses’ follow-up and interventions were sufficient and on time”; “my nurses took care to use protective equipment, such as gloves and masks”; “hospital service was generally good”; “I prefer the hospital again and recommend it to others.” The patients were asked to answer each question on a five-point scale, including very dissatisfied, dissatisfied, average, satisfied, and very satisfied, to evaluate the satisfaction of both groups of patients. The Cronbach’s alpha coefficient for this scale was 0.880 [32].

### Statistical analyses

Data were presented as mean ± SD. and analyzed using the SPSS software (version 26.0). Continuous variables were compared between the two groups using Student’s t-test, while discontinuous variables were compared using the Chi-square test (*P* < 0.05).

## Results

### Baseline information of patients received different nursing strategies

The regular group included 16 males and 29 females with an average age of 72.27 ± 7.92 years, while the ERAS group enrolled 17 males and 28 females with an average age of 71.44 ± 6.99 years. There were no significant differences in age, gender, and BMI between the two groups (Table 2). Patients who received regular nursing care were mainly diagnosed with femoral bone necrosis (55.56%), neck fracture of the femur (28.89%), and a minority with other diagnoses (15.56%). Patients in the ERAS group were also diagnosed with femur bone necrosis (60.00%), femoral neck fractures (26.67%), and a minority with other diagnoses (13.33%). Additionally, a history of disease, smoking, and drinking showed insignificant differences between the two groups.

**Table 2:** Baseline information of study subjects

	regular	ERAS	*P*-value
Age	72.27 ± 7.92	71.44 ± 6.99	0.603
Gender (M/F)	16/29	17/28	0.827
BMI	23.26 ± 2.37	23.00 ± 2.65	0.628
Disease history			
Hypertension (n,%)	20, 44.44	21, 46.67	0.832
Diabetes (n,%)	7, 15.56	9, 20	0.581
Coronary heart disease (n,%)	4, 8.89	3, 6.67	0.694
Clinical diagnosis			0.908
Femur bone necrosis (n,%)	25, 55.56	27, 60	
Neck fracture of femur (n,%)	13, 28.89	12, 26.67	
Other (n,%)	7, 15.56	6, 13.33	
Smoking (n,%)	11, 24.44	13, 28.89	0.634
Drinking (n,%)	9, 20	10, 22.22	0.796

### The recovery conditions of patients received different nursing strategies

As shown in Table 3, patients receiving the ERAS nursing strategy were found to have an earlier time of first aerofluxus, getting out of bed, and first defecation, and moved a farther distance relative to patients receiving a regular nursing strategy. Additionally, patients receiving ERAS had the urinary and drain tubes removed earlier than those receiving regular nursing strategies.

**Table 3:** Postoperative recovery conditions of study subjects

	Regular	ERAS	*P*-value
First aerofluxus (h)	35.54 ± 5.84	29.91 ± 5.64	< 0.001
Primary getting out of bed (h)	71.34 ± 7.93	35.86 ± 7.28	< 0.001
Moving distance (m)	8.37 ± 1.49	14.39 ± 2.10	< 0.001
First defecation (h)	60.57 ± 5.06	50.62 ± 4.79	< 0.001
removal of urinary tube (h)	35.19 ± 5.45	18.61 ± 3.21	< 0.001
Removal of drain tube (h)	48.63 ± 5.80	37.94 ± 6.45	< 0.001

**Fig. 1:**
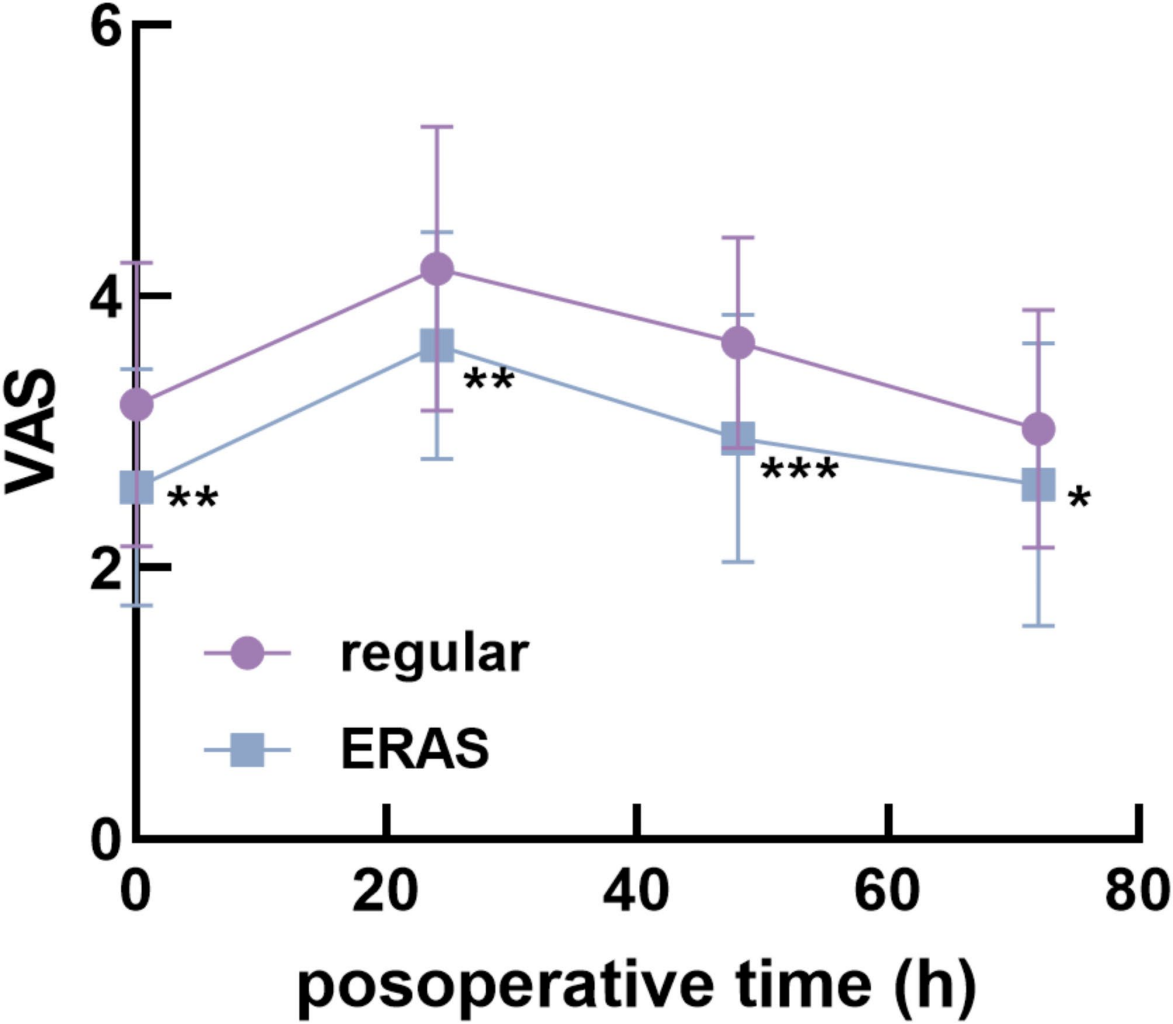
Postoperative VAS scores of patients receiving different nursing strategies. The VAS of the ERAS group was significantly lower than that of the regular group after postoperative 24 h (*P* < 0.01), 48 h (*P* < 0.001), and 72 h (*P* < 0.05). ^*^P < 0.05, ^**^P < 0.01, ^***^P < 0.001

**Fig. 2:**
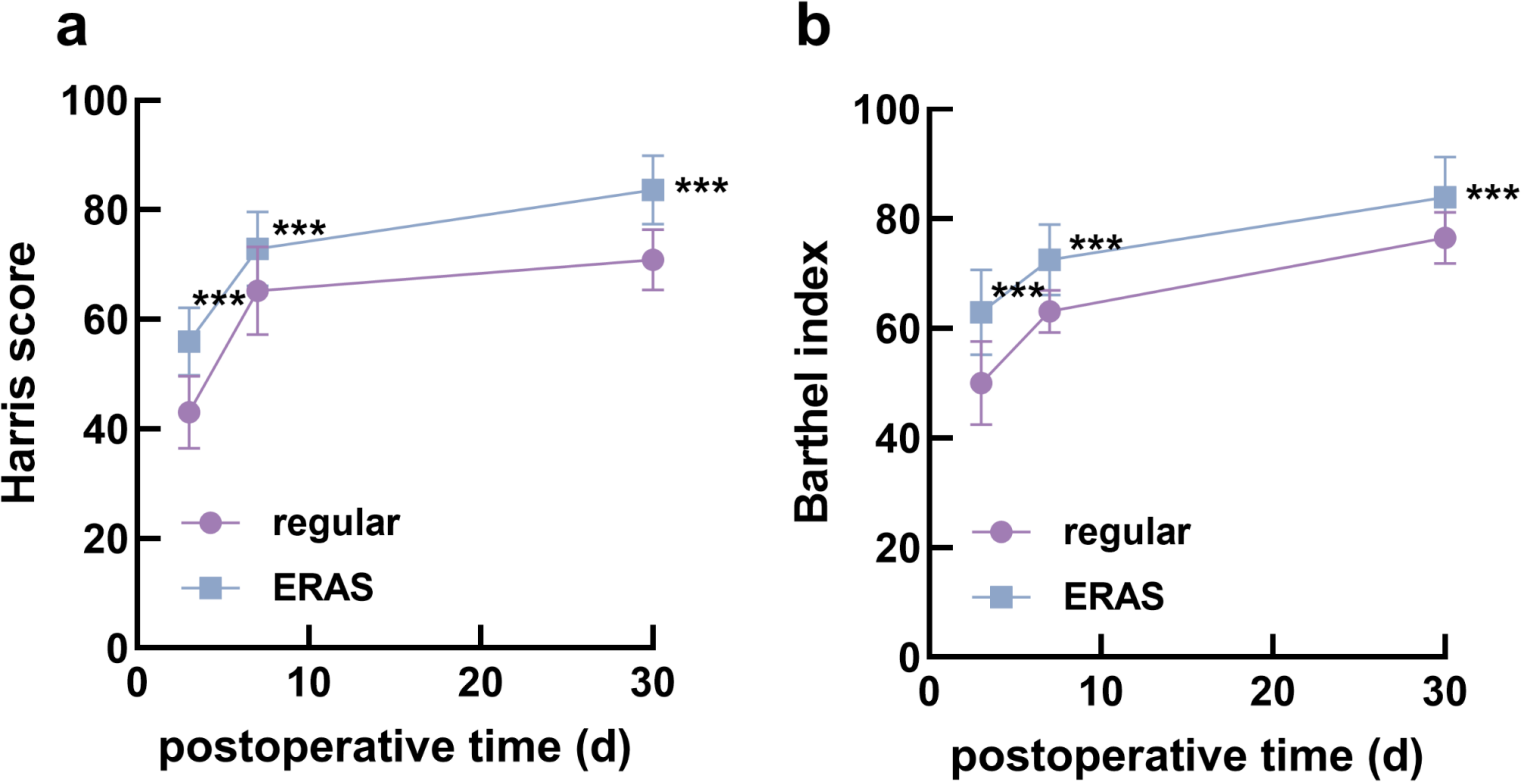
Postoperative Harris score (for evaluating hip function, **a**) and Barthel index (for evaluating ability to daily life, **b**) of two groups. **a**, Harris score indicated that patients receiving ERAS care strategy had faster hip function recovery than those receiving regular care (*P* < 0.001). **b**, Barthel index showed that the ability of daily living of patients who received ERAS care strategy recovered faster than those who received regular care (*P* < 0.001). ^***^P < 0.001

In contrast, the VAS scores of the ERAS group were much lower than those of the regular group at 24, 48, and 72 h postoperatively (Fig. 1). The hip function and activities of daily living of patients who received the ERAS nursing strategy also recovered more quickly than those who received regular nursing, as indicated by the Harris scores (Fig. 2a) and Barthel index (Fig. 2b).

### The hospitalization and complications of patients who received different nursing strategies

The ERAS nursing strategy can reduce hospitalization expenses and shorten the duration of hospitalization for patients. Patients receiving the ERAS nursing strategy also showed higher satisfaction, with only one not satisfied and there were three “not satisfied” in-patients receiving regular nursing (Table 4). There were no significant differences in satisfaction between the two groups.

**Table 4:** The hospitalization and complication of study subjects

	regular	ERAS	*P*-value
Hospitalization expenses (ten thousand yuan)	6.76 ± 0.66	6.24 ± 0.48	< 0.001
Hospitalization duration (d)	13.44 ± 2.04	8.33 ± 1.83	< 0.001
Satisfaction			0.220
very satisfied	25	30	
satisfied	10	12	
in general	7	2	
not satisfied	3	1	
very dissatisfied	0	0	
Complications			0.008
Deep vein thrombosis	3	0	
Urinary system infection	4	1	
Pulmonary infection	3	0	
Joint stiffness	2	0	
constipation	10	3	
uroschesis	3	1	

Complications included deep vein thrombosis (*n* = 3), urinary system infection (*n* = 4), pulmonary infection (*n* = 3), joint stiffness (*n* = 2), constipation (*n* = 10), and uroschesis (*n* = 3). Patients receiving the ERAS nursing strategy only showed complications, such as urinary system infection (*n* = 1), constipation (*n* = 3), and uroschesis (*n* = 1), and the incidence rate was lower than that in patients receiving regular nursing.

## Discussion

THA is a common treatment for end-stage hip joint disease, particularly in older adult patients and those who do not respond to conservative treatment. To maximize patient satisfaction and surgical outcomes while reducing costs and optimizing resources, ERAS was progressively introduced in THA surgery as a multidisciplinary approach tailored to each patient [21, 33]. Patients receiving ERAS got out of bed earlier after surgery and moved a longer distance than those receiving regular nursing. The walking distance in the ERAS group on the sixth day after surgery was 1000 m, while that in the conventional care group was only 625 m, and the difference was significant [28]. In ERAS, pain management was optimized, which effectively controled postoperative pain and provided a premise for postoperative activity [34]. Additionally, optimized pain management reduced the application of opioids, inhibiting the occurrence of urinary retention; therefore, the removal of the urinary and drainage tubes of patients receiving ERAS occurred earlier, increasing the convenience of activities [35]. Postoperative aerofluxus and defecation are also critical indices for evaluating the recovery of gastrointestinal function. The fasting time in ERAS was shortened before surgery, and the postoperative supplementation of water and liquid diet was also earlier than with regular nursing, which avoids discomfort, such as electrolyte loss and intestinal mucosal dysfunction, caused by long-term fasting and drinking [36]. Moreover, earlier postoperative activity in patients receiving ERAS could also accelerate blood circulation, improve blood oxygen, and promote the discharge of intestinal contents [37].

After THA, obesity increases the perioperative incidence and mortality rates and is associated with a higher risk of readmission and reoperation [21]. However, there was no statistically significant difference in the BMI between the two patient groups in this study. Additionally, this study found that patients receiving the ERAS strategy had earlier time to first airflow, ambulation, and first defecation, longer distance traveled, and earlier removal of the catheter and drainage tube than those receiving the conventional care strategy. This is similar to the results obtained by Gotz et al. [28] and Li et al. [38]. Additionally, compared with traditional nursing, the ERAS nursing strategy can reduce postoperative hospitalization time, hospitalization costs, and functional recovery time (first exhaust time, defecation time, oral dosage, postoperative walking ability, etc.) in patients with gastric cancer [39]. Zhang et al. also confirmed that the ERAS nursing strategy is superior to conventional nursing in patients undergoing cholangiopancreatography (ERCP) for the treatment of bile duct stones and can effectively accelerate patient recovery and reduce the incidence of complications [40]. A systematic review and meta-analysis indicated that ERAS can shorten the hospitalization, exhaustion, defecation, activity, and drainage tube removal times of prostate cancer patients with RALP/LRP compared with conventional care [41]. Moreover, this study found that the VAS scores of patients who received the ERAS nursing strategy after surgery were much lower than those in the conventional group. A systematic review and meta-analysis indicated that ERAS could reduce patient VAS scores and improve ROM, SF-36 BP, and SF-36 PF scores [25]. Another systematic review and meta-analysis showed that ERAS significantly reduced the VAS score and incidence of complications in older adult patients undergoing joint replacement surgery compared with non-ERAS [42]. Furthermore, emergency cesarean section patients receiving the ERAS nursing strategy had lower VAS scores during the initial walking and resting periods on days 0 and 1 than the control group [43]. This indirectly reflected the reliability of the results.

Additionally, this study used the Harris score and Barthel index to evaluate hip function and ability in daily life. Patients who received the ERAS nursing strategy showed better hip function and ability to perform activities of daily living. The “fast track management” guided by the ERAS concept has significant advantages in early surgery for intertrochanteric fractures in older adult patients. The Harris and Barthel scores in the ERAS group were higher than those in the conventional group at one and two weeks after surgery; however, at one month after surgery, there was no significant difference in the VAS and Harris scores between the two groups [44]. Zhu et al. conducted a propensity score-matched analysis and found that the Harris scores of the ERAS nursing strategy in older adult patients undergoing surgery for intertrochanteric fractures were higher than those in the control group at one and three months, but the difference was not significant at six months, suggesting that this approach can promote early recovery of hip joint function in patients [45]. ERAS improves the postoperative rehabilitation training of patients and sets daily activity goals. Moreover, the training strategy and daily goals were adjusted according to the status of the patients, which increased joint motion, reduced joint adhesion, effectively prevented muscle atrophy, and hence improved the patients’ ability to perform activities of daily living [46]. Additionally, ERAS can improve the postoperative satisfaction score and Barthel index score of patients with diabetic foot ulcers (DFU), and promote postoperative recovery [47]. In summary, ERAS has demonstrated significant advantages in various nursing fields and is of great significance for improving patient recovery speed, reducing medical costs, optimizing medical resources, and other aspects.

From the perspective of postoperative complications, deep vein thrombosis is a severe complication of THA due to blood hypercoagulation, blood stasis, and vascular intimal injury during surgery [48–50]. Aging of the enrolled patients also increased the risk of deep vein thrombosis. Three months after surgery is a high-incidence period for deep vein thrombosis; therefore, the follow-up period for complications was prolonged to the time after discharge. ERAS supplemented the training of the ankle and humeral quadriceps after anesthesia, which effectively promoted lower limb venous return and reduced the incidence of deep vein thrombosis. Uroschesis is another major postoperative complication of THA in the present study. The inhibition of urinary flexion by opioids is the main cause of uroschesis [51]. ERAS optimized pain management, where a multi-mode analgesic scheme was adopted and opioids were reduced, thereby reducing the risk of uroschesis [52]. A previous study found that multimodal analgesia reduced the inflammatory response in patients with colorectal cancer undergoing radical surgery by lowering the level of the inflammatory marker IL-6 [53]. A key component of multimodal analgesia is preemptive analgesia, which not only improves joint function and pain control but also promotes faster postoperative functional recovery [54]. Additionally, regional anesthesia and other multimodal analgesics may alleviate pain and promote functional recovery by blocking the activation and sensitization of the peripheral and central nervous systems caused by injury. Local anesthetics themselves have anti-inflammatory properties, which can reduce inflammatory reactions [55]. In vitro studies and animal model experiments have confirmed that local anesthetics can reduce ectopic neuronal discharge, decrease the expression of cytokines and other inflammatory mediators, and reduce neutrophil activation [56, 57].

According to the aforementioned ERAS optimization, patient satisfaction significantly improved. Recent studies have also included psychological support and health education in the ERAS nursing strategy, which promotes all aspects of postoperative rehabilitation of patients [58–60]. Therefore, the ERAS content can be further optimized in terms of mental health. However, the ERAS nursing strategy involved the optimization of surgery, anesthesia, psychology, and nutrition. Therefore, surgeons, anesthesiologists, nurses, and other allied health professionals require professional training and strict standards to ensure desired nursing effectiveness in clinics [61].

Previous studies have shown that the safety and effectiveness of ERAS in joint replacement surgery are primarily achieved by strengthening patient education, providing perioperative nutritional support, optimizing anesthesia, preventing infection and venous thromboembolism, and optimizing analgesic regimens [21, 24, 62]. This study found that ERAS nursing strategies promote patient recovery by optimizing nursing measures compared with routine care. In terms of pain management, the ERAS group received preventive analgesia before surgery, short-term continuous spinal anesthesia during surgery, local infiltration analgesia, and oxycodone and conventional analgesics after surgery. In terms of nutritional support, the ERAS nursing strategy group had a special nutrition team that assessed the nutritional status of the patients, formulated a personalized nutritional intervention plan before surgery, calculated the energy intake of the patients at different stages after surgery, and supplemented nutrition as needed. Therefore, ERAS can accelerate the recovery process and improve patient satisfaction by optimizing anesthesia, developing multimodal analgesia programs, and providing personalized nutritional support.

Overall, ERAS has potential benefits for the postoperative care of various human diseases. Therefore, the implementation of ERAS nursing strategies requires careful preoperative preparation, intraoperative care, postoperative care, nutritional support, and rehabilitation training. In terms of preoperative preparation, it is necessary to conduct a comprehensive preoperative evaluation of the patient, advise them to pay attention to preoperative matters and encourage them to perform preoperative rehabilitation exercises. In terms of intraoperative concerns, it is necessary to closely monitor the patient’s vital signs to ensure safety during the procedure, and to use appropriate anesthesia methods and pain management measures to reduce the stress response of the patient to surgery. In terms of postoperative care, it is necessary to closely observe the vital signs, degree of pain, defecation, and exhaustion of patients; provide drug and non-drug pain relief measures; reduce the pain and discomfort of patients; promote early action and recovery; encourage patients to get out of bed; and provide rehabilitation training as soon as possible after surgery to reduce the occurrence of complications and accelerate the rehabilitation process. Nutritional support and rehabilitation training provide patients with personalized nutritional support programs, rehabilitation programs, and rehabilitation training skills to help them recover their physical function gradually.

### Limitations and future directions

This study has several limitations. First, the small sample size and large number of women included in this study may have led to a decrease in the reliability, validity, and representativeness of the research results, as well as gender bias. In future research, we will expand the sample size and consider the gender balance to further validate the results of this study. Second, this study was conducted using a single-blind research design, with only the surgeons knowing the grouping; however, the data were evaluated by the entire nursing team, resulting in selection bias. To enhance the robustness of our research design, we plan to implement a double-blind approach in future studies. Additionally, subjective questionnaires were used to evaluate satisfaction without considering psychological measurement issues, which may have led to inaccurate information, sample bias, or an inability to gain a deeper understanding. Once again, the rapid updating of professional nursing knowledge makes it difficult for nursing staff to keep up with the latest developments. Therefore, we must strengthen continuing education and establish knowledge-updating mechanisms. Finally, ERAS nursing strategies can be further optimized, such as reducing postoperative physical activity and functional impairment, reducing postoperative cognitive dysfunction, and identifying high-risk patients with complications [21]. Therefore, in the future, we will include more cases, adopt a multicenter design, and optimize the ERAS protocol to further extend the results of this study.

## Conclusion

This single-blind randomized controlled trial demonstrated that patients undergoing ERAS demonstrated earlier first flatus, ambulation, and defecation; greater distance of ambulation after initial ambulation; and earlier removal of urinary and drainage tubes compared with the regular care group. In addition, ERAS can significantly reduce postoperative pain, improve the Harris score and Barthel index, shorten the length and cost of hospital stay, reduce the incidence of complications, and improve patient satisfaction. Taken together, this study demonstrates the safety and efficacy of ERAS nursing strategies in terms of clinical outcomes. The ERAS nursing strategy is recommended for older adult patients undergoing THA to improve their postoperative recovery.

## Data Availability

Data supporting this study may be requested from the corresponding author upon reasonable request.
